# PB.38: In the context of overdiagnosis, does size matter?

**DOI:** 10.1186/bcr3538

**Published:** 2013-11-08

**Authors:** S Bhuva, I Haigh, M McMahon, B Dall, D Dodwell, N Sharma

**Affiliations:** 1 Leeds Teaching Hospitals, Leeds, UK

## Introduction

The Marmot Review showed that although breast screening saves lives, it is harmful through overdiagnosis - treating cancers that would not otherwise have ever become clinically apparent. Currently, there is no size threshold for recalling screening patients with calcifications. Our aim was to assess whether a minimum size threshold would reduce overdiagnosis.

## Methods

We conducted a retrospective review of 375 screening patients with microcalcifications over 24 months. We assessed all patients with pure calcifications ≤10 mm documenting core biopsy, final histology and treatment.

## Results

Sixty-one cases of microcalcifications ≤10 mm: eight benign, 40 *in situ *cancers and 13 invasive cancers. This group was subcategorised into calcifications: 0 ≤5 mm (24 patients) and 5 ≤10 mm (37 patients). In the 0 ≤5 mm group, there were 16 *in situ *(low-grade, one; intermediate grade, seven; high grade, eight) and two invasive cancers (G2 ductals ER/PR^+^Her2^- ^node-negative). In the 5 ≤10 mm group, there were 24 *in situ *(low-grade, three; intermediate grade, 12; high grade, nine) and 11 invasive cancers (four G1ER^+^Her2^- ^node-negative, six G2ER^+^Her2^-^, one triple-negative). One of these six cases was node-positive (micrometastasis) and one G3ERPR^+^Her2^- ^node-negative. All underwent wide local excision, and all but one patient with invasive carcinoma received radiotherapy.

## Conclusion

Recalling focal clusters of microcalcifications (<10 mm) identified a high rate of cancers: 66% (40/61) *in situ *and 21% (13/61) invasive. With regards to overdiagnosis: 51% (27/53) of cancers were low/intermediate-grade DCIS or G1 invasive and 49% (26/53) were high-grade DCIS or invasive G2/3. Therefore size is not a key factor in reducing overdiagnosis.

